# Assessing the Global Impact of Ambient Air Pollution on Cancer Incidence and Mortality: A Comprehensive Meta-Analysis

**DOI:** 10.1200/GO-24-00402

**Published:** 2024-10-24

**Authors:** Paolo Boffetta, Monireh Sadat Seyyedsalehi

**Affiliations:** Paolo Boffetta, MD, MPH, Department of Medical and Surgical Sciences, University of Bologna, Bologna, Italy, Stony Brook Cancer Center, Stony Brook University, Stony Brook, NY, Department of Family, Population and Preventive Medicine, Stony Brook University, Stony Brook, NY; and Monireh Sadat Seyyedsalehi, MSc, Department of Medical and Surgical Sciences, University of Bologna, Bologna, Italy

## To the Editor:

Several systematic reviews and meta-analyses were published in recent years aimed at quantifying the association between outdoor air pollution and cancer, particularly lung cancer.^1-8^ These meta-analyses were helpful to assess the causal nature of these associations and their magnitude and contributed to establish guidelines on the basis of exposure levels whose estimated effects are considered acceptable.^9^

Ramamoorthy et al^10^ recently published in the Journal a meta-analysis of the effect of air pollution on cancer incidence and mortality. Their main result was a relative risk (RR) of 1.04 (95% CI, 1.02 to 1.05) for cancer incidence. Unfortunately, the study contains methodologic issues that warrant further consideration and discussion.

In the primary analyses, the authors combined risk estimates for exposure to four different pollutants (PM2.5, PM10, NO_2_/NOx, and O_3_). This approach may lead to biased results because of the differing impacts of the pollutants as there is evidence that the four pollutants do not exert a similar effect on cancer risk.^9^

In addition, the authors combined risk estimates for different cancers. This issue is similar to the one discussed above since there is evidence that the carcinogenic effect of air pollution depends on the type of cancer.^6,9,11^ In addition, for many types of cancer, such as lung cancer, there are different histologic types which might have different etiologies, and it would be preferable to analyze them separately whenever possible.

Furthermore, in several instances, the authors included in the meta-analysis several risk estimates from the same study. This error exaggerates the contribution of studies reporting multiple results compared with those reporting a single result, independent of other characteristics such as precision and validity of the risk estimates.

In addition, the authors failed to rescale their results to a common exposure contrast. Even if results of different studies addressed the same pollutant and the same cancer, they might compare different exposure intervals. An increase of 10 μg/m^3^ would yield, everything else being equal, a higher RR than an increase of 5 μg/m^3^. Previous meta-analyses rescaled all results according to a common exposure contrast.^1-5,7,8^

An additional potential source of bias derives from the fact that several studies included in the review did not adjust for potential confounding factors, including tobacco smoking, obesity, and occupational carcinogenic exposures. This limitation of the underlying studies, and its implications on the summary risk estimates, should be discussed.

For these reasons, although the overall results of the meta-analysis reported by Ramamoorthy et al^10^ provides valuable insights into the relationship between air pollution and cancer, addressing these methodologic limitations would strengthen the conclusions. The authors also provided secondary results stratified by cancer type and by pollutant, thus somehow addressing some of the criticisms mentioned above. However, the inconsistency in exposure contrast between studies was not addressed. Meta-analyses are technically straightforward, yet they require careful design and execution to avoid errors which would generate wrong estimates. As a suggestion, the authors might consider analyzing the pollutants separately in future work to account for their differing effects on cancer risk on the basis of consistent exposure contrasts.
